# Supplementary material for: Somatic mutations in early metazoan genes disrupt regulatory links between unicellular and multicellular genes in cancer
Source: eLife. 2019 Feb 26;8:e40947. doi: 10.7554/eLife.40947 (PMC6402835; doi:10.7554/eLife.40947)
Supplement: Transparent reporting form [file elife-40947-transrepform.pdf]

eLIFE
1st Floor
24 Hills Road
Cambridge CB2 1JP, UK
P 01223 855340
W elifesciences.org
T @elife

eLife's transparent reporting form

We encourage authors to provide detailed information *within their submission* to facilitate the interpretation and replication of experiments. Authors can upload supporting documentation to indicate the use of appropriate reporting guidelines for health-related research (see EQUATOR Network), life science research (see the BioSharing Information Resource), or the ARRIVE guidelines for reporting work involving animal research. Where applicable, authors should refer to any relevant reporting standards documents in this form.

If you have any questions, please consult our Journal Policies and/or contact us: editorial@elifesciences.org.

Sample-size estimation

- You should state whether an appropriate sample size was computed when the study was being designed
- You should state the statistical method of sample size computation and any required assumptions
- If no explicit power analysis was used, you should describe how you decided what sample (replicate) size (number) to use

Please outline where this information can be found within the submission (e.g., sections or figure legends), or explain why this information doesn't apply to your submission:

Not applicable. We have conducted a survey of publicly available cancer genomics data, not a 'case-control' study.

Replicates

- You should report how often each experiment was performed
- You should include a definition of biological versus technical replication
- The data obtained should be provided and sufficient information should be provided to indicate the number of independent biological and/or technical replicates
- If you encountered any outliers, you should describe how these were handled
- Criteria for exclusion/inclusion of data should be clearly stated
- High-throughput sequence data should be uploaded before submission, with a private link for reviewers provided (these are available from both GEO and ArrayExpress)

Please outline where this information can be found within the submission (e.g., sections or figure legends), or explain why this information doesn't apply to your submission:

Gene expression patient samples with a low correlation to other samples and evident outliers in PCA were excluded from the analysis. Exclusion criteria for specific genes based on the unreliability of mutation calls is explained in the methods sections.

All high-throughput sequence data were obtained from already public available resources, The Cancer Genome Atlas. No new high-throughput data was generated in this study.

Statistical reporting

- Statistical analysis methods should be described and justified
- Raw data should be presented in figures whenever informative to do so (typically when N per group is less than 10)
- For each experiment, you should identify the statistical tests used, exact values of N, definitions of center, methods of multiple test correction, and dispersion and precision measures (e.g., mean, median, SD, SEM, confidence intervals; and, for the major substantive results, a measure of effect size (e.g., Pearson's r, Cohen's d)
- Report exact p-values wherever possible alongside the summary statistics and 95% confidence intervals. These should be reported for all key questions and not only when the p-value is less than 0.05.

Please outline where this information can be found within the submission (e.g., sections or figure legends), or explain why this information doesn't apply to your submission:

The statistical methods, p-values and other metrics are presented at relevant locations in the manuscript, as well as in the Methods section.

(For large datasets, or papers with a very large number of statistical tests, you may upload a single table file with tests, Ns, etc., with reference to sections in the manuscript.)

Group allocation

- Indicate how samples were allocated into experimental groups (in the case of clinical studies, please specify allocation to treatment method); if randomization was used, please also state if restricted randomization was applied
- Indicate if masking was used during group allocation, data collection and/or data analysis

Please outline where this information can be found within the submission (e.g., sections or figure legends), or explain why this information doesn't apply to your submission:

Not applicable. Samples did not need to be allocated into groups, since the groups were defined based on tissue type (tumour or normal) and tumour type.

Additional data files ("source data")

- We encourage you to upload relevant additional data files, such as numerical data that are represented as a graph in a figure, or as a summary table
- Where provided, these should be in the most useful format, and they can be uploaded as "Source data" files linked to a main figure or table
- Include model definition files including the full list of parameters used
- Include code used for data analysis (e.g., R, MatLab)
- Avoid stating that data files are "available upon request"

Please indicate the figures or tables for which source data files have been provided:

eLife Sciences Publications, Ltd is a limited liability non-profit non-stock corporation incorporated in the State of Delaware, USA, with company number 5030732, and is registered in the UK with company number FC030576 and branch number BR015634 at the address 1st Floor, 24 Hills Road, Cambridge CB2 1JP | August 2014

All datasets were obtained from public repositories and are thus already publicly available. Gene expression and point mutation data was obtained from The Cancer Genome Atlas; the gene regulatory network from PathwayCommons, gene cell line dependency scores from Project Achilles, mutation and CNA information of cell lines from the Cancer Cell Line Encyclopaedia (CCLE) (https://portals.broadinstitute.org/ccle) and IC50 values of cancer cell lines after drug treatment from the Genomics of Drug Sensitivity in Cancer database. The sources and versions of the data are indicated throughout the manuscript and in the Methods sections.

All data generated as a result of our downstream analysis and represented in the figures is available in the supplementary files of the manuscripts. The code needed to reproduce all analysis and figures is available at https://github.com/cancer-evolution/Evolutionary-analysis-of-somatic-mutations-in-cancer
